# The Efficacy of Ultraviolet Radiation in Decontaminating Dropped Bone Fragments in Orthopedic Surgery

**DOI:** 10.7759/cureus.87698

**Published:** 2025-07-10

**Authors:** Owais A Qureshi, Siddhartha Sinha, Neel Aggarwal, Asif Iqbal, Neetu Shree, Javed Jameel, Sandeep Kumar

**Affiliations:** 1 Department of Orthopedics, Hamdard Institute of Medical Sciences and Research, New Delhi, IND; 2 Department of Orthopedics, Malda Medical College and Hospital, Malda, IND; 3 Department of Microbiology, Hamdard Institute of Medical Sciences and Research, New Delhi, IND

**Keywords:** anti-infective agents, bone graft decontamination, decontamination, dropped bone fragment, dropped bone graft, dropped sample, povidone-iodine, saline solution, ultraviolet rays

## Abstract

Introduction: The dropping of bone after harvesting for grafting compromises sterility. Often, washing the bone with an antiseptic solution, harvesting a new autograft, or using an allograft is the only options. This study aims to determine the effectiveness of ultraviolet (UV) radiation in restoring the sterility of contaminated bone fragments and compare it to other commonly used methods for decontamination.

Methods: The excised bone was divided into five equal parts. One part was sent for culture without any treatment in a sterile environment. The other four parts were dropped sequentially on the theater floor, retrieved in a sterile manner, treated with either normal saline (NS), 5% povidone betadine solution (PBS), or a UV chamber, and sent for Gram stain and culture to assess growth.

Results: It was observed that 23.6% (n = 9) of the samples dropped on the floor and, when cultured without treatment, showed Coagulase-negative Staphylococci (11.1%, n = 4) and aerobic spore bearers (13.8%, n = 5). After treatment with UV, 84.2% (n = 32) of the samples did not show any growth, and 86.8% (n = 33) of the samples did not show growth with PBS. There was a significant difference in growth between samples treated with NS and those treated with PBS or in the UV chamber. PBS solution was superior in the decontamination of bone.

Conclusion: Treatment in a UV chamber for five minutes was effective in restoring sterility to bone fragments dropped on the floor, but its effects on viability are not known. Future studies are needed to explore the role of UV radiation in the decontamination of bone and its maintenance of vitality.

## Introduction

The operating room is a sterile environment; however, there are situations when sterility can be compromised. One such scenario that commonly occurs is the dropping of instruments, bone fragments, or soft tissue graft after harvesting [1,2]. In such situations, it becomes imperative to restore the sterile nature of the tissue or instrument to prevent postoperative infection and graft failure. The incidence of autograft contamination ranges from 0% to 70% [1,3]. Following a fall of the autograft, the most common options are to wash the graft with an antiseptic solution, harvest a new autograft, or use an allograft [1,4]. Decontamination of the graft to restore sterility can be done by various methods, including washing with antiseptic solutions such as chlorhexidine and betadine, or autoclaving the graft. These methods are either inefficient and time-consuming or leave doubts regarding the viability of the graft [3-8].

Ultraviolet radiation (UVR) has been hypothesized to induce dimerization of DNA/RNA as a function of UVR intensity. This dimerization can lead to the inactivation of microorganisms, leading to decontamination. Thus, UVR has the potential to provide a convenient and cost-effective method for decontamination [9]. With the COVID-19 pandemic, the use of UVR to decontaminate objects has become more common. There has been growing interest in the use of UVR for decontamination in various settings, including food sanitation and healthcare. Memic et al. reported that far UV-C is effective in reducing contamination of patient transport chairs, physical therapy equipment, and seats [10]. Griffin et al. irradiated colonies of *Enterococcus faecium, Escherichia coli*, *Pseudomonas aeruginosa*, and *Staphylococcus aureus* and reported that Far-UV chamber light is effective in decontaminating bacterial vegetative cells and endospores [11]. Maugeri et al., in their systematic review of 25 studies, also concluded that UV light technologies have the potential to reduce hospital-acquired infections; however, their efficacy varies depending on the application, pathogen type, and healthcare setting. They also emphasized that further research is needed to optimize its use in healthcare settings [12]. Sicks et al. demonstrated that Far-ultraviolet C (UVC) radiation has the potential to decontaminate human and porcine corneal endothelial cells without compromising endothelial cell viability [13].

To the best of our knowledge, no studies are comparing the efficacy of UVR to commonly used methods for the decontamination of dropped harvested bone. We hypothesized that UVR could be an effective method for decontamination of dropped bone fragments. This study aimed to determine the effectiveness of UVR in restoring the sterility of a contaminated harvested bone fragment and compare it to other commonly used methods in such situations.

Part of the abstract has been published in the conference proceedings for the Delhi Orthopaedic Association Conference 2024 (DOACON 2024) in the Journal of Clinical Orthopaedics and Trauma (available as 10.1016/j.jcot.2024.102662), which took place in November 2024.

## Materials and methods

Study details and inclusion criteria

This prospective nonrandomized study was conducted at a tertiary care center between June 2023 and December 2023 after obtaining appropriate ethical approval. All patients who had been planned for a hemireplacement arthroplasty, total hip arthroplasty, total knee arthroplasty, or corrective osteotomy where the excised fragment of bone would have been otherwise discarded/destroyed were included in the study. Samples from patients unwilling to give consent, patients with a history of septic or tubercular arthritis in the joints, patients with any active infection or on antitubercular therapy in the last two years, and patients with a history of chronic osteomyelitis or any other chronic infection in the body were excluded from the study. Four surgeons conducted the surgery; two surgeons performed the preparation and decontamination of the samples.

Technique of preparing, contaminating, and decontaminating the bone fragments in the operating theater

**Figure 1:**
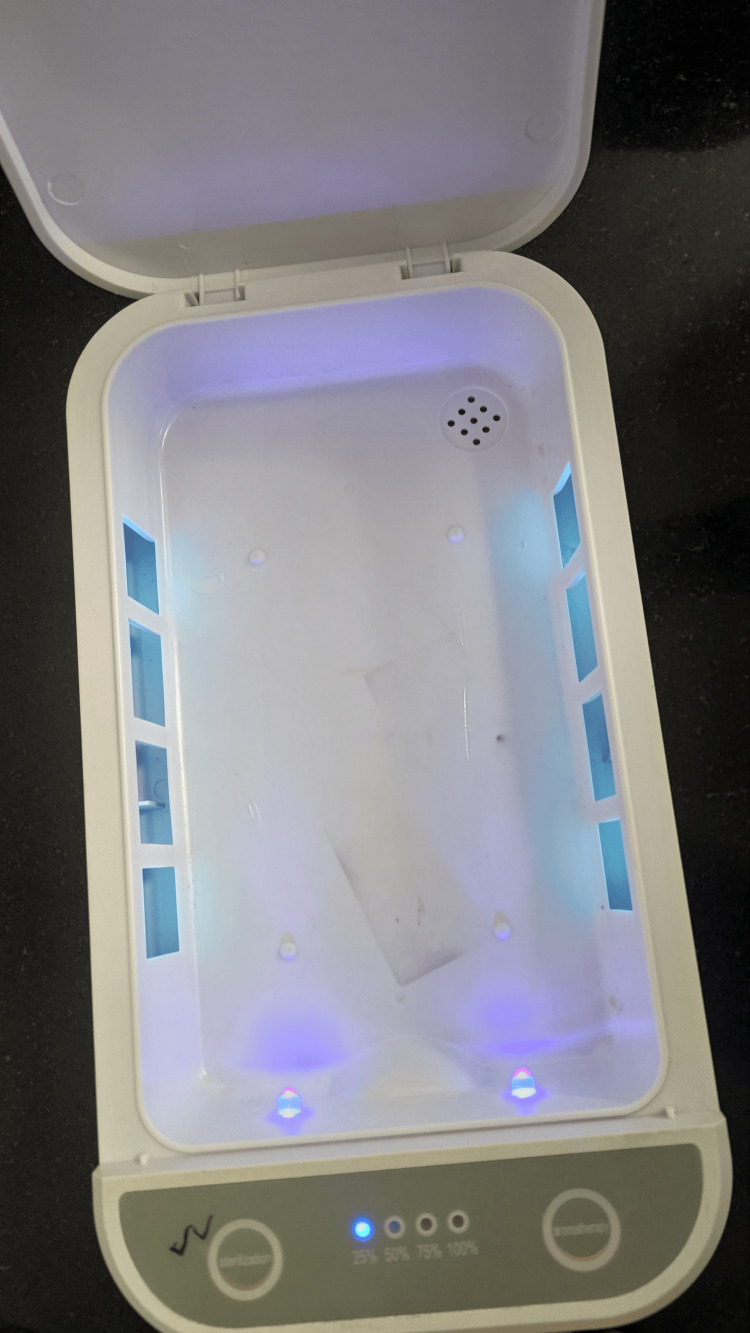
A commercially available ultraviolet chamber with a microscopic slide used for the study

The excised bone (femoral head, distal femur, and proximal tibia) was divided into five equal-sized parts of equal sizes of 1 x 1 x 1 cm using a straight 10 mm osteotome on the operating table. Part 1 (P1) was sent for culture immediately after preparation without any treatment in a sterile environment. The other four parts were dropped one by one on an area within 0.5 m of the surgical table, within an area of 10 x 10 cm from a height of about 30 cm and allowed to stay in contact with the floor for 10 seconds, to simulate the maximum time from dropping to retrieving the graft [14]. The fragments were dropped one by one and were retrieved in a sterile manner. Part 2 (P2) was sent for culture without treatment with any solutions. Part 3 (P3) was sent for culture after treatment with washing in normal saline (NS). The sample was soaked in the solution for one minute with gentle agitation of the container, after which it was transferred to a container with NS for transport and culture. Part 4 (P4) was sent for culture after treatment with a 5% betadine solution for one minute, with gentle agitation of the container, and was transported in a standardized manner. Part 5 (P5) was sent for culture after treatment within a UVC that emits UVR of 260-280 nm with a running cycle of five minutes (Figure 1).

**Figure 2:**
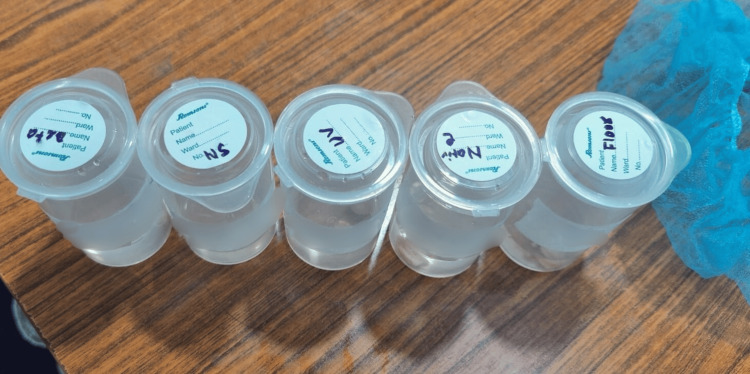
All samples transferred to containers with normal saline and transported for culture after appropriate treatment for decontamination

For preparing the UVC, after thorough cleaning of the inner chamber with Sterillium and wiping it using sterile precautions with NS, an initial cycle without any samples was run to ensure disinfection. The sample was then mounted on a sterile glass slide to ensure UVR penetration on all sides. The samples were immediately placed in containers with NS for further microbiological processing with standard methods (Figure 2).

**Figure 3:**
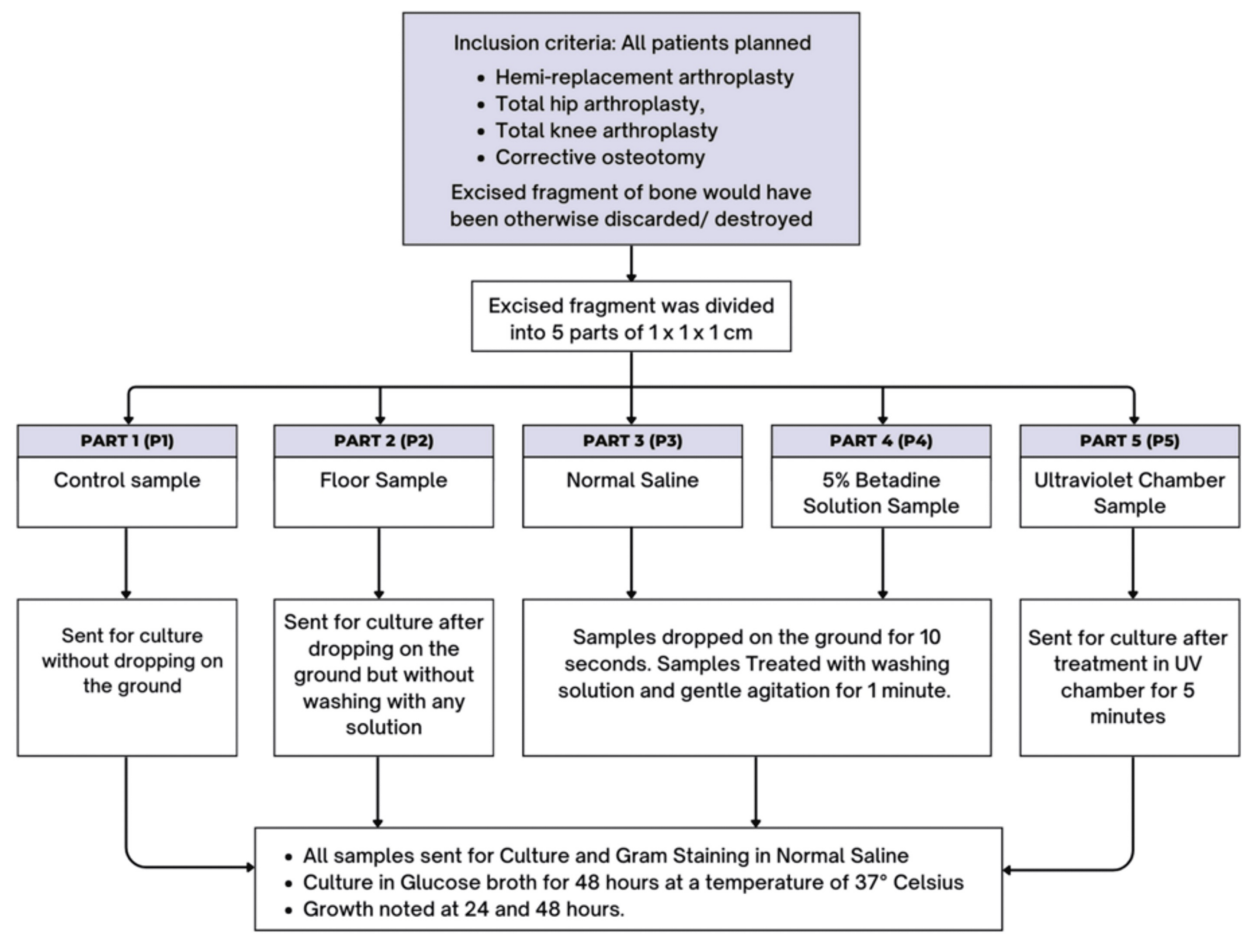
Summary of the study protocol followed

The samples were cultured in glucose broth for 48 hours at a temperature of 37°C after initial Gram staining and plating on blood and MacConkey agar so as not to miss any significant growth, even if a minimal bacterial load was present in the sample. Culture plates and broths were further observed for growth at 24 and 48 hours, and final reporting was done at the end of 48 hours. The culture plates were used after standard quality tests for media, i.e., sterility test and observing typical growth of standard American Type Culture Collection Quality Control strains, i.e., *S. aureus* 25923, *Staphylococcus epidermidis* 12228, *Streptococcus pyogenes* 19615, *Shigella flexneri* 12022, and *E. coli* 25922. Liquid media was used as it promotes rapid growth. At no point in the study were these fragments reimplanted or reintroduced into any patients, and they were discarded. No changes to the floor cleaning protocol were requested before the surgeries. Figure 3 shows a summary of the study.

Statistical analysis

The data collected was entered into Microsoft Excel 2019 (Microsoft Corporation, Redmond, WA) and was provided a unique number. Final statistical analysis was performed using the Statistical Package for the Social Sciences, version 25 (IBM Inc., Armonk, NY). Proportions, percentages, mean, and standard deviation were calculated for descriptive analysis. The chi-square test was used to assess any significant difference between the decontamination methods.

## Results

A total of 38 surgeries were performed during the study period. The left side (55.3%, n = 21) was more commonly operated on than the right side (44.7%, n = 17). The average age of the population was 58.84 ± 14.13 years. Taking account of each surgery performed, 55.3 % (n = 21) were women and 44.7% (n = 17) were men. The most common diagnoses were neck of femur fractures (50%, n = 19), followed by osteoarthritis of the knee (34.2%, n = 13). The most frequently performed surgery was hemireplacement arthroplasty (42.1%, n = 16).

**Figure 4:**
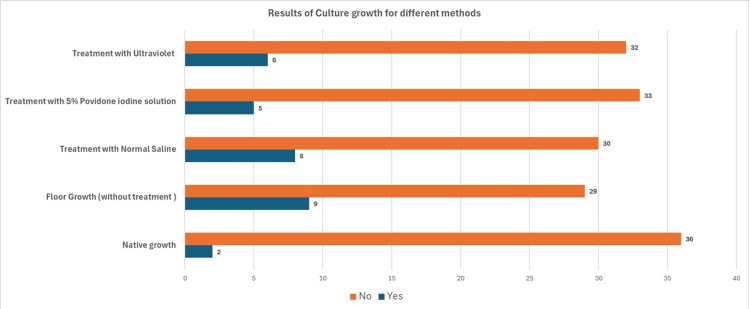
Culture positivity obtained by treatment of the dropped pieces from different methods

**Figure 5:**
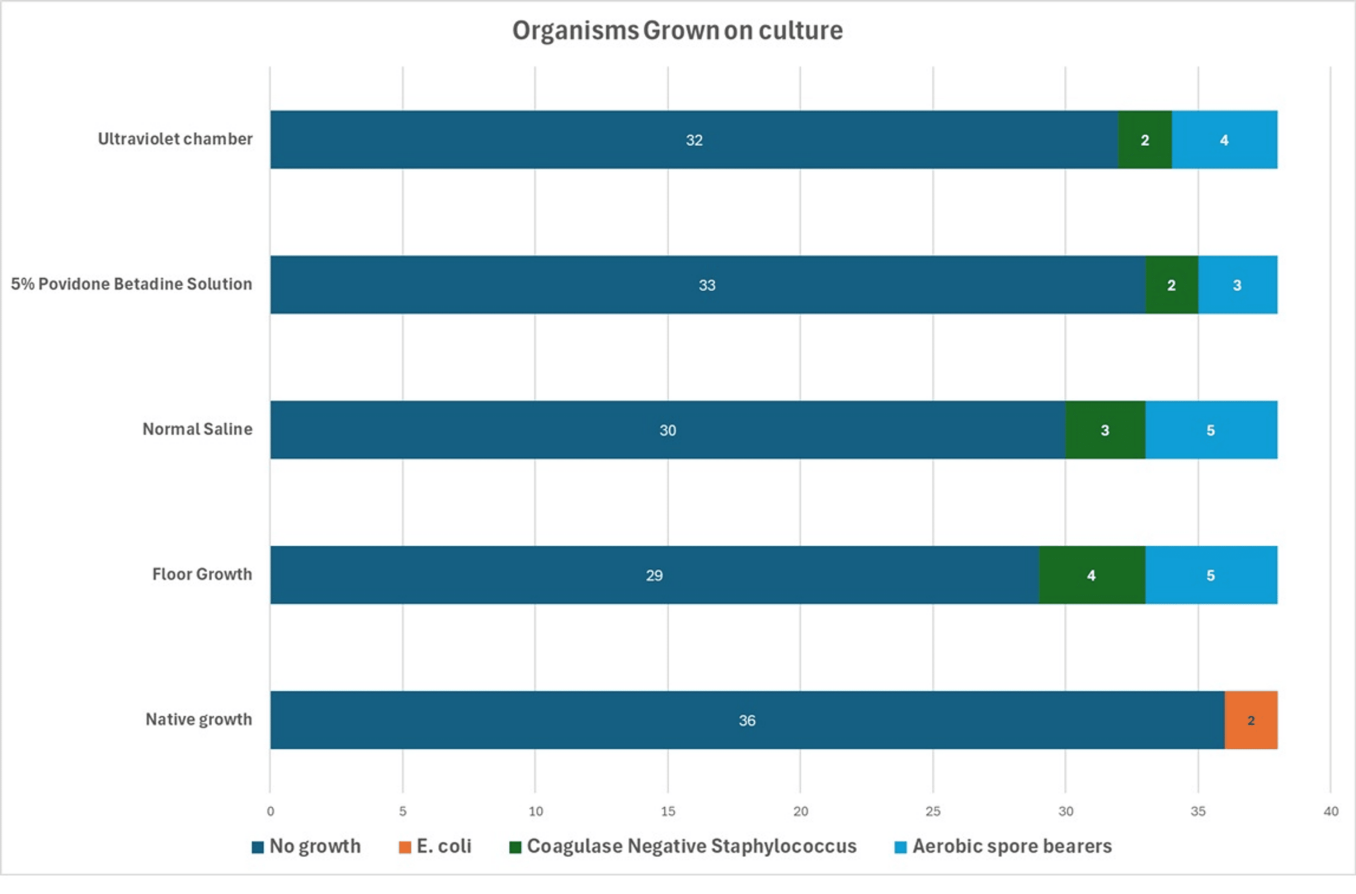
Details of organisms grown on culture

Out of 38 samples, 97.4% (n = 36) of the native culture samples did not show any growth. Two native cultures (5.5%) grew *E. coli*; 23.8% (n = 9) of the samples dropped on the floor and cultured without any treatment showed growth. The growth organism in the untreated dropped sample consisted of Coagulase-negative Staphylococci (CoNS) (10.5%, n = 4) and aerobic spore bearers (ASBs) (13.1%, n = 5). Five samples (13.2%) treated with povidone betadine solution (PBS) showed growth consisting of CoNS (5.3%, n = 2) and ASB (7.9%, n = 3); 21.1% (n = 8) samples washed with NS showed growth consisting of ASB (13.15%, n = 5) and CoNS (7.9%, n = 3); 84.21% (n = 32) samples, after treatment in the UV chamber, did not show any growth, and 15.7% (n = 6) reported growth. Of these, 5.2% (n = 2) grew CoNS and 10.5% (n = 4) grew ASB (Figures 4, 5).

There was a significant difference in the growth of microorganisms in samples that were not treated with any method compared to those treated with NS, 5% betadine solution, or in the UVC after dropping on the floor. There was also a significant difference in growth between samples treated with NS and those treated with PBS or UVC. Significant differences in growth were observed between the samples treated with UVC and those treated with PBS, indicating that PBS was superior to UVC for decontamination (Table 1).

**Table 1:** Chi-square test comparing the different methods used for decontamination of the dropped fragments Statistically significant value considered for p < 0.05

Parameters	Chi-square	p value
No treatment for the dropped graft		
Normal saline	16.76	0.001
Betadine	38	0.001
UV chamber	29.57	0.001
Wash with normal saline compared to betadine and UV chamber treatment of the dropped fragments		
Betadine	16.76	0.001
UV chamber	21.59	0.001
Comparison of betadine solution wash and UV chamber treatment		
UV chamber	29.57	0.001

## Discussion

The dropping of a harvested bone graft intraoperatively is a catastrophic event. Restoring sterility and viability of the graft becomes of utmost importance. Most surgeons do not record or inform patients of such events, and the long-term effects of reusing such grafts are not well-documented [15]. Many studies have explored many methods to restore the sterility and viability of these dropped fragments. Setting guidelines and protocols for such an event is difficult due to many factors, including hospital infrastructure, the virulence of the normal flora in the operating theater, as well as the availability of appropriate instruments and machines, to name a few. UV light has been shown to have sterilization effects; however, its use for restoring sterility in dropped grafts has not been explored. This study successfully reports that a treatment with a UV chamber can restore sterility to a dropped bone graft. Its efficacy is better than treatment with NS but not better than PBS.

Altınayak et al. reported that 10% PBS was an effective method of decontamination without affecting bone cell viability. They also reported no difference in growth in sodium hypochlorite and nonintervened groups [5]. Pommer et al. reported that 1% PBS was effective in reducing the colony-forming units (CFUs) [16]. Barbier et al. also reported the least growth with 10% PBS for hamstring grafts dropped on the floor [4]. Bauer et al. [6] reported negative cultures for all samples that were treated with dry PBS (10% iodine allowed to dry for 15 minutes). This study also shows that the results of treating the dropped fragment with 5% PBS were better than providing treatment to the fallen bone fragment, as well as treatment with NS. PBS works by releasing iodine, which has been shown to be chemically toxic to microorganisms [17]. Hooe and Steinberg reported that there were no histological changes in the dropped bone fragments of rats after exposure to 10% PBS [18]. Soyer et al. also reported preferring the use of 10% PBS due to its low toxicity and ease of availability [19]. Yazdi et al. reported the least positive cultures following decontamination by 0.4% chlorhexidine or 10% PBS in dropped rabbit bones [20]. In contrast to these findings, Mat-Salleh et al. [8] reported high rates of positive cultures treated with PBS and alcohol but low positive growth with chlorhexidine. Soyer et al. reported that 10% PBS was effective in decontamination. In a systematic review by Mortazavi et al. [3], they concluded that a five-minute bath in 10% PBS, followed by a one-minute bulb irrigation lavage with NS, successfully decontaminated bone grafts and maintained cellular viability.

Pommer et al. reported that exposure to a 1% chlorhexidine solution to produce negative cultures while maintaining bone graft vitality involved a 15-second wash with the solution. They also reported that 1% chlorhexidine solution also provided a similar decrease in CFUs but was more effective in some cases than PBS for decontaminating bone grafts [16]. A study by Bauer et al. [6] compared different methods of decontamination of bone graft and found that autoclaving and chlorhexidine treatment did not leave viable cells. Centeno et al. reported that, unlike PBS and chlorhexidine, bacitracin solution was not toxic to fibroblasts and recommended against the use of PBS [21]. This study did not study the effects of cell viability following decontamination methods. Bruce et al. reported that a five-minute wash with 10% PBS, followed by an NS wash, effectively decontaminated and retained the viability of the graft in 51% of the samples. They also reported that 4% chlorhexidine treatment did not leave viable cells, and physical scrubbing of the graft decreased the overall cell viability irrespective of the solution used [22]. Hirn et al. reported the least positive cultures following high-pressure NS wash but did not assess cell viability [23]. Kang et al. reported that following the dropped graft 90% used low-pressure lavage to decontaminate the graft [15]. Molina et al. also reported that 4% chlorhexidine was most effective in decontaminating dropped tendon grafts, followed by a neomycin/polymyxin solution, with positivity rates of 4%-6%, respectively. Similar results for contaminated tendon grafts have also been reported by Plante et al. [24].

Common microorganisms isolated from various studies included *S. epidermidis* (24%-97%), Bacillus (4%-20%), and *E. coli* (17%) [3]. The culture growth patterns for the floor in this study were similar to those noted by the infection control committee for the institution, with most being CoNS and ASB. This pattern is also similar to those reported by Alomar et al., Bruce et al., Hirn et al., and Molina et al. [14,22,23,25]. Mat-Salleh et al. also reported growing CoNS and ASB, which were similar to their control group [8]. The source of Bacillus is proposed to be from clean clothes worn in the theater and Staphylococcus via the patient or surgeon [26]. Most other authors also reported growth consistent with the floor fauna of their operating room. The growth of the floor organism has not been shown to predict the growth after dropping the graft for bone or tissue by some authors [2,4]. The most common organism isolated in cultures was *S. epidermidis* [4,5,14]. Other authors have reported the growth of *P. aeruginosa*, *Klebsiella pneumoniae*, and *E. coli* [5]. Nondetection of *S. aureus* in this study was possibly due to its low CFU and overgrowth by other bacteria [14]. Bacillus was reported to be resistant to the effects of PBS and alcohol, but sensitive to chlorhexidine by Mat-Salleh et al. [8]. In contrast, Bruce et al. reported a 100% positive culture of Bacillus despite treatment with PBS, 4% chlorhexidine, or isopropyl alcohol/2% chlorhexidine gluconate, regardless of the duration of exposure or mechanical treatment [22]. A possible explanation for the *E. coli* growth in the uncontaminated samples was that these were skin contaminants. Both samples were from hip hemiarthroplasty surgeries from geriatric patients and could be skin contaminants.

The role of UV radiation has not been reported in the decontamination of dropped bone grafts. UVR induces DNA damage and consequent replication of pathogens [27]. It has been shown to decrease Gram-negative rods but not *Clostridioides difficile* and vancomycin-resistant enterococci [28]. Kovach et al. reported the successful use of UV-C in decontaminating surfaces. They reported a 7.1% and 4.4% positivity rate for Gram-positive and Gram-negative organisms, respectively. As a consequence, they reported an overall decrease in nursing home-acquired infection rates [29]. Yang et al. also reported a decrease in bacterial colonies after UV irradiation for 15 minutes [30]. Our study indicates that PBS is superior to using a UV chamber for disinfection of the dropped bone graft.

This study was a growth promotion study, and hence, CFUs were not calculated after a positive culture. This study also did not calculate the cell viability of the graft after disinfection. The study did not conduct a comparison with other antimicrobial agents, such as chlorhexidine. The study was limited to bone and not soft tissue grafts, which have been dropped on the theater floor.

There is a lot of variation in the way a dropped bone graft in the operating room is decontaminated, and there is no uniform protocol that has been established that restores sterility while maintaining viability. Ultraviolet C radiation is being regularly used in both daily and healthcare settings for decontamination; however, its role in the decontamination of bone grafts is not documented.

## Conclusions

Our study concludes that treatment in a UV chamber for five minutes is superior to washing with NS for decontamination; however, washing the dropped bone fragments with PBS was shown to be superior to treatment in a UV chamber in restoring sterility. The use of UV for bone graft decontamination shows promise, and we encourage future studies to explore the role of UV radiation on cell viability as well as the time used for disinfection.
